# The complete mitochondrial genome of *Acanthaspis geniculata* (Hsiao, 1976) (Hemiptera: Reduviidae) and its phylogenetic analyses

**DOI:** 10.1080/23802359.2026.2705001

**Published:** 2026-08-25

**Authors:** Wanting Liu, Jiahao Chen, Jiakun Lin, Ping Wang, Xi Cheng

**Affiliations:** ^a^ College of Forestry, Fujian Agriculture and Forestry University, Fuzhou, China; ^b^ Fujian Colleges and Universities Engineering Research Institute of Conservation and Utilization of Natural Bioresources, College of Forestry, Fujian Agriculture and Forestry University, Fuzhou, China; ^c^ College of Plant Protection, Fujian Agriculture and Forestry University, Fuzhou, China

**Keywords:** *Acanthaspis geniculata*, Reduviidae, mitochondrial genome, phylogenetic analysis

## Abstract

The genus Acanthaspis (Hemiptera: Reduviidae) comprises predatory insects of ecological importance, yet mitochondrial genomic resources for this group remain limited. Here, we present the complete mitochondrial genome of *Acanthaspis geniculata* (Hsiao, 1976). The mitogenome is 16,226 bp in length and contains the standard set of 37 mitochondrial genes (13 protein-coding genes, 2 rRNA genes, and 22 tRNA genes), along with a control region (D-loop). The nucleotide composition is A (40.4%), T (29.6%), C (17.9%), and G (12.1%), with an overall AT content of 70.0%. Phylogenetic analyses based on partitioned mitochondrial datasets strongly support the monophyly of the subfamily Reduviinae and reveal that *A. geniculata* forms a close sister-group relationship with *A. ruficeps* and *A. pedestris*. This study provides a fundamental mitogenomic resource for future evolutionary and systematic studies of the Reduviidae.

## 1. Introduction

**Figure 1.**
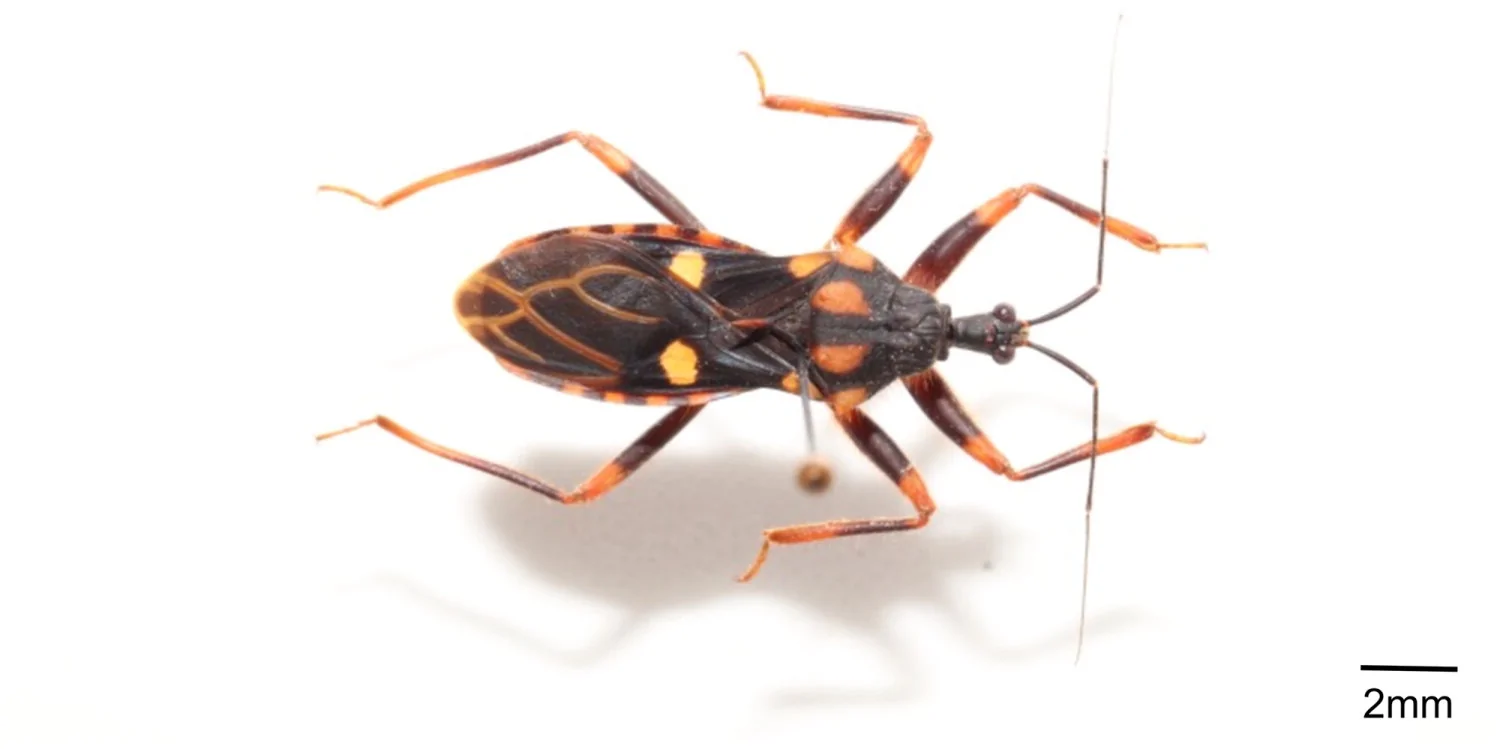
Adult habitus of *Acanthaspis geniculata* (Hsiao, 1976). Standard body length of the specimen is 16 mm, collected from Wenshan, Yunnan Province, China (104°14′46″E, 23°22′5″N) on August 2024. Photographed by Wanting Liu. Scale bar = 2 mm.

*Acanthaspis*, the second largest genus within the subfamily Reduviinae, exhibits a primary distribution across the Oriental and the Afrotropical regions. (Cao 2011) Fourteen species of this genus have been recorded from China, among which *Acanthaspis geniculata* (Hsiao, 1976) (Hemiptera: Reduviidae) is included (Cao et al. 2014). *A. geniculata* is characterized by two key morphological features: circular spots present on the corium and clavus of the forewings, adults with a body color ranging from dark brown to black, while the prothoracic pronotum is trapezoidal and bears wrinkles on the posterior region (Figure 1) (Xiao 1976; Cao 2011). The nymphs of *A. geniculata* are characteristically gregarious, generally univoltine, and undergo overwintering as either eggs or fifth-instar nymphs (Cao et al. 2014; Kou et al. 2017). *A. geniculata* is an insect natural enemy that acts as a predator of diverse insect taxa, including Lepidopteran larvae, *Tenebrio molitor* (yellow mealworms), and ants. Among congeneric species, notable examples include *A. collaris* and *A. cincticrus* (which prey on *Lepidopteran larvae* and ants) (Kou et al. 2017), *A. petax* Stål (Which feeds primarily on termites; Odhiambo 1958), and *A. pedestris* Stål (known to prey on *Lepidopteran larvae*; Ambrose 1986).

Gaining an understanding of the biology and ecology of insect natural enemies is of great significance for biological control and the sustainable development of the ecological environment (Beus et al. 2024). To date, 75 mitochondrial genomes (mitogenomes) of assassin bugs from only 11 subfamilies have been reported (Ye et al. 2021). The limited sampling of the mitogenome at higher categories hinders a deep understanding of mitogenome evolution and reduviid phylogeny. Current research on the family Reduviidae has focused primarily on the description of new taxonomic units, gregarious behavior, and studies on biodiversity (Weirauch 2006; Swanson 2017; Galvão et al. 2024; Masonick et al. 2025), whereas reports on phylogenetic studies related to this family have been rather limited in number. Through the analysis of mitochondrial genes, the phylogenetic relationships between *A. geniculata* and its congeneric species can be elucidated. Furthermore, in terms of supplementing molecular evidence for the faunal evolution of Reduviinae in China, However, the phylogenetic relationships within Chinese Reduviinae remain to be further explored based on molecular markers.

## 2. Materials

Adult specimens of *A. geniculata* were collected from Wenshan, Yunnan Province, China (104°14′46″E, 23°22′5″N) using sexual pheromone traps, and were subsequently subjected to complete mitochondrial genome sequencing for molecular phylogenetic analyses. The Key Laboratory of Integrated Pest Management in Ecological Forests, Fujian Agriculture and Forestry University (URL: https://lxy.fafu.edu.cn; contact person: Xi Cheng; email: schengxi@163.com) served as the repository for voucher specimen LC-202408.

## 3. Methods

### 3.1. Mitochondrial genome assembly and annotation

The leg of an adult *A. geniculata* individual was employed for the extraction of total genomic DNA, which was subsequently purified using the TruSeq DNA Sample Preparation Kit (Vazyme, Fuzhou, China) and the QIAquick Gel Extraction Kit (Qiagen, Hilden, Germany). The quality and concentration of the extracted DNA were assessed using a NanoDrop spectrophotometer (Thermo Fisher Scientific, Waltham, MA, USA). Subsequently, DNA sequencing was conducted on an Illumina HiSeq 2500 platform (Illumina, San Diego, CA, USA). The total sequencing data obtained for the mitochondrial genome was 6 Gb. After initial filtration of the 58,852,938 raw reads to remove low-quality bases and adapters, we obtained 55,807,474 high-quality whole-genome clean reads. Then, mitochondrial homologous sequences were enriched *via* reference alignment, yielding a final set of 1,176,336 mitochondrial-specific clean reads for mitogenome assembly. These clean reads were assembled using MitoZ (Meng et al. 2019) and metaSPAdes software (Nurk et al. 2017). The optimal assembly result was selected as the final mitogenome, which is a single circular sequence of 16,226 bp with a contig N50 of 16,226 bp and no ambiguous bases (Ns = 0). The completeness of the mitogenome was confirmed by successful circularization and the presence of all 37 typical mitochondrial genes (13 PCGs, 22 tRNAs, and 2 rRNAs). The MITOs web server (Matthias et al. 2013) was employed for annotating the assembly results using the reference sequence of *A. pedestris* (GenBank accession number: NC_069624). The obtained genomic sequence data have been deposited in the NCBI database under accession number PX310134. The genome map was visualized using the Proskee (Grant et al. 2023). Additionally, transfer RNA (tRNA) genes were predicted using tRNAscan-SE software (Lowe and Eddy 1997).

### 3.2. Phylogenetic analysis

Aiming to achieve a more thorough insight into the taxonomic status of *A. geniculata*, we built a phylogenetic tree using the complete mitochondrial genomes of 21 additional Cimicomorpha species (retrieved from NCBI BLAST, https://blast.ncbi.nlm.nih.gov) and *A. geniculata* itself, with *Adrisa magna* (Hemiptera: Cydnidae; GenBank accession number: NC_042429.1) as the outgroup (Table 1). Complete mitochondrial genomes were subjected to alignment *via* the MUSCLE software (Edgar 2022). Subsequently, ambiguously aligned sites and gaps were filtered out from the multiple sequence alignments using Gblocks (Castresana 2000; Dereeper et al. 2008), yielding a conserved core dataset for downstream phylogenetic analyses. To improve phylogenetic inference, a partitioning strategy was adopted dividing the dataset into PCGs and rRNA regions, for which the best-fit nucleotide substitution models were independently selected using ModelFinder (Kalyaanamoorthy et al. 2017) based on the Bayesian Information Criterion (BIC), identifying GTR+F + R3 for the PCGs partition and GTR+F + I + R3 for the rRNA partition. Based on these partitioned models, a Maximum Likelihood (ML) tree was constructed using IQ-TREE (Minh 2020), with branch support rigorously evaluated using the ultrafast bootstrap approximation (UFBoot) with 4,000 replicates and the Shimodaira–Hasegawa approximate likelihood-ratio test (SH-aLRT) with 1,000 replicates. Additionally, a Bayesian Inference (BI) tree was reconstructed using MrBayes v3.2.7 (Ronquist et al. 2012), employing two independent Markov Chain Monte Carlo (MCMC) runs for 2,000,000 generations, sampling every 1,000 generations, and discarding the first 25% of sampled trees as burn-in to ensure convergence. To conclude, iTol v7 (https://itol.embl.de/) was used for visualizing the resulting phylogenetic tree.

**Table 1.** Information on the mitogenomes of the species used in this study.

Family	Subfamily	Species	GenBank no.	References
Cydnidae	Cydninae	*Adrisa magna*	NC_042429.1	Unpublished
Notonectidae	Notonectinae	*Notonecta amplifica*	MZ305077.1	Li et al. (2022)
		*Notonecta montandoni*	NC_045886.1	Minh et al. (2020)
Reduviidae	Peiratinae	*Peirates atromaculatus*	NC_026670.1	Zhao et al. (2015)
		*Peirates lepturoides*	NC_026672.1	Unpublished
		*Peirates fulvescens*	NC_026669.1	Zhao et al. (2015)
	Reduviinae	*Platymeris biguttatus*	PP198281.1	Unpublished
		*Acanthaspis geniculata*	PX310134.1	This study
		*Acanthaspis ruficeps*	NC_037369.1	Unpublished
		*Acanthaspis pedestris*	NC_069624.1	Unpublished
		*Acanthaspis cincticrus*	NC_037735.1	Zhao et al. (2019)
		*Inara alboguttata*	NC_037736.1	Gong et al. (2018)
		*Reduvius gregoryi*	NC_037746.1	Liu et al. (2019)
	Triatominae	*Triatoma boliviana*	NC_086924.1	Unpublished
		*Triatoma huehuetenanguensis*	OR611932.1	Cigarroa-Toledo et al. (2024)
		*Triatoma infestans*	NC_035547.1	Unpublished
		*Triatoma dimidiata voucher*	MT757848.1	Cigarroa-Toledo et al. (2024)
	Harpactorinae	*Agriosphodrus dohrni*	NC_015842.1	Li et al. (2011)
		*Velinus nodipes*	NC_037738.1	Unpublished
	Phymatinae	*Amblythyreus gestroi*	NC_037733.1	Chen et al. (2018)
		*Phymata americana*	NC_036011.1	Li et al. (2017)
Miridae	Mirinae	*Lygus pratensis*	NC_037926.1	Tan et al. (2018)
		*Apolygus lucorum*	NC_023083.1	Wang et al. (2014)

Note: These species used in this study are the complete mitochondrial genomes.

## 4. Results

The assembled mitochondrial genome of *A. geniculata* was supported by high sequencing coverage across the entire genome, with an average sequencing depth of 1050.8×, a maximum depth of 1748×, and a minimum depth of 99× (Figure S1).

**Figure 2.**
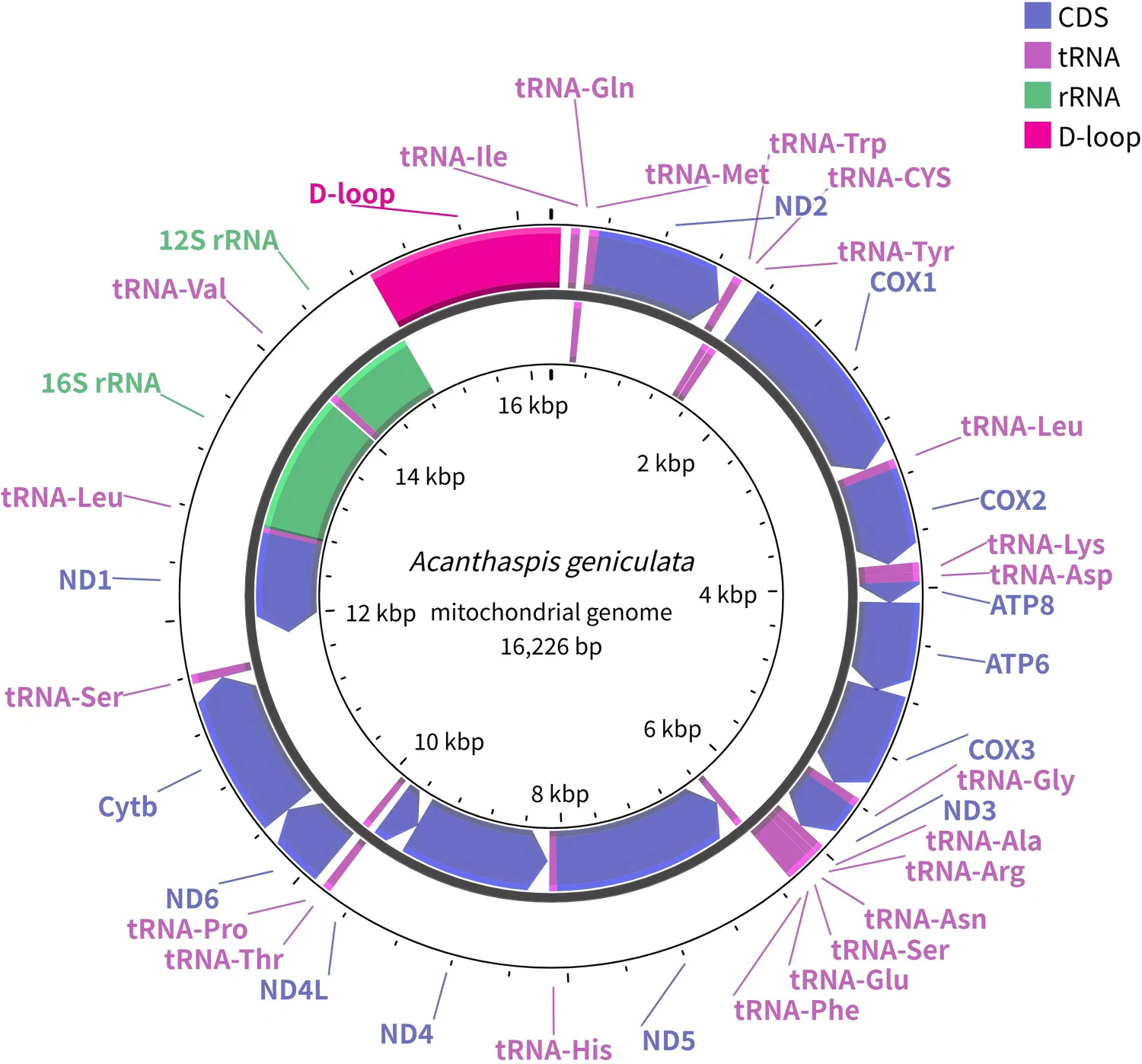
Mitogenome map of the mitochondrial genome of *Acanthaspis geniculata* (GenBank: PX310134). The inner circle indicates the gene length, and the colors on the external circle indicate different genes and regions. The arrows represent the direction of transcription; genes encoded on the heavy and light strands are shown inside and outside the circle, respectively.

A total of 58,852,938 raw reads were yielded by Illumina HiSeq 2500. Raw reads were filtered using Genomics Workbench to remove adapter sequences and low-quality reads, and mitochondrial homologous sequences were subsequently enriched, which resulted in a total of 1,176,336 mitochondrial-specific clean reads. Local BLAST search of de novo assembled contigs using the complete mitochondrial genome of *Acanthaspis pedestris* (NC_069624.1) identified the complete mitochondrial genome of *A. geniculata*. The complete mitochondrial genome (DDBJ/EMBL/GenBank accession number: PX310134.1) is a double-stranded and circular molecule with 16,226 bp in length, containing 13 PCGs, 22 tRNA genes, 2 rRNA genes, and one D-loop (Figure 2). The nucleotide composition is A (40.4%), T (29.6%), C (17.9%), and G (12.1%) with a pronounced AT bias (70%), while the GC content is 30.0%. The light strand encodes 9 PCGs (ND2, COX1, COX2, ATP8, ATP6, COX3, ND3, ND6 and CYTB), 14 tRNA genes (tRNA-Leu, tRNA-Lys, tRNA-Asp, tRNA-Gly, tRNA-Ala, tRNA-Arg, tRNA-Asn, tRNA-Ser, tRNA-Glu, tRNA-Thr, tRNA-Ile, tRNA-Met, tRNA-Trp and tRNA-Ser) and D-loop, whereas heavy strand encodes the other 4 PCGs (ND5, ND4, ND4L, ND1), 8 tRNA genes (tRNA-Gln, tRNA-Cys, tRNA-Tyr, tRNA-Phe, tRNA-His, tRNA-Pro, tRNA-Leu, tRNA-Val) and 2 rRNA (rrnL, rrnS). Among PCGs, ATG is used as the start codon for COX1, ATP6, COX3, ND6, CYTB, ND4L, ND4 and ND1. ATA for ND2 and ATP8, ATT for COX2 and ND3, GTG for ND5. TAA is used as the stop codon for ND2, COX2, ATP8, ND5, ND6, ND4, ND4L and ND1. TAG for ATP6 and CYTB. TA for COX3. An incomplete stop codon (a single T codon) was found in one PCG (COX1). The rrnL and rrnS genes are 1248 bp and 767 bp in length, respectively. Gene overlaps are present, notably a 7 bp overlap between ATP8 and ATP6, and a 7 bp overlap between ND4 and ND4L (Table S1)

**Figure 3.**
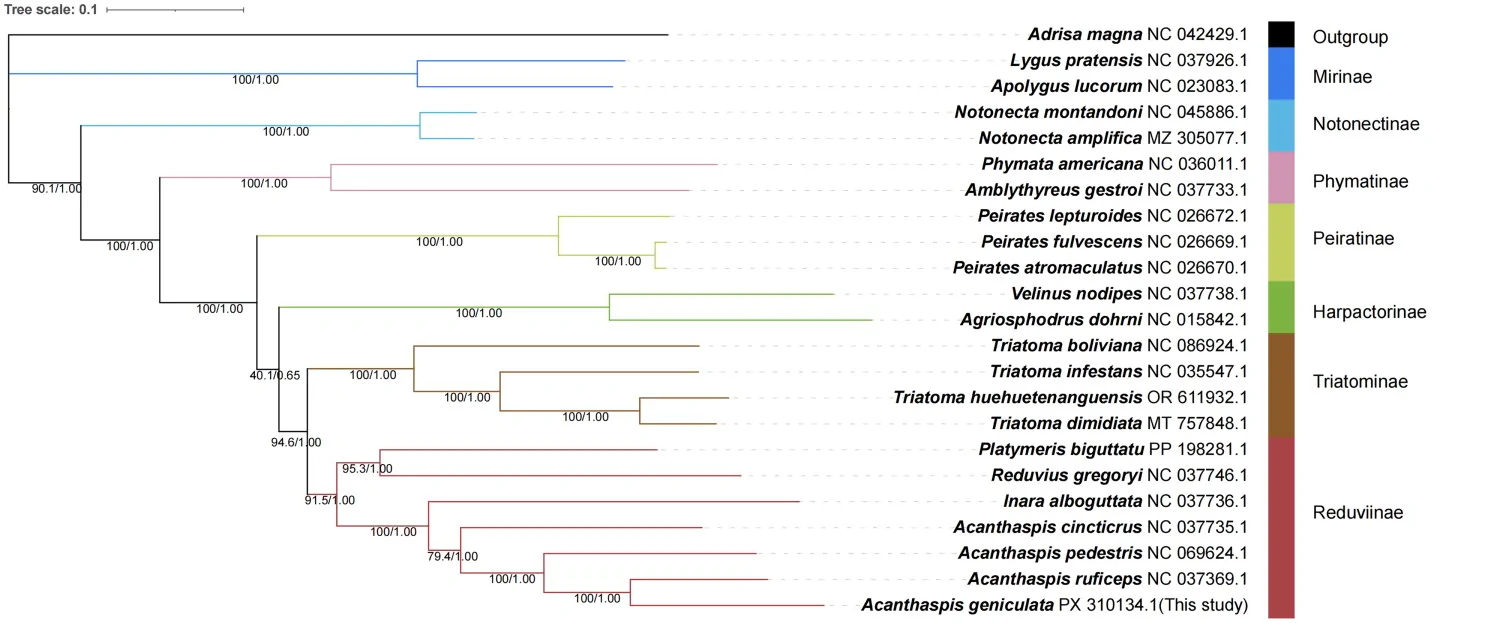
Phylogenetic relationships within Reduviidae and related taxa reconstructed by maximum likelihood (ML) and Bayesian inference (BI) analyses based on a concatenated dataset of 13 mitochondrial protein-coding genes (PCGs) and 2 rRNA genes. Nodal support values are presented as ML bootstrap percentages (BS) and Bayesian posterior probabilities (PP), respectively, in the format BS/PP. the following sequences were used: NC_042429.1 (Unpublished), MZ305077.1 (Li et al. 2022), NC_045886.1 (Minh et al. 2020), NC_026670.1 (Zhao et al. 2015), NC_026672.1 (Unpublished), NC_026669.1 (Zhao et al. 2015), PP198281.1 (Unpublished), PX310134.1 (This study), NC_037369.1 (Unpublished), NC_069624.1 (Unpublished), NC_037735.1 (Zhao et al. 2019), NC_037736.1 (Gong et al. 2018), NC_037746.1 (Liu et al. 2019), NC_086924.1 (Unpublished),OR611932.1 (Cigarroa-Toledo et al. 2024), NC_035547.1 (Unpublished), MT757848.1 (Cigarroa-Toledo et al. 2024), NC_015842.1 (Li et al. 2011), NC_037738.1 (Unpublished), NC_037733.1 (Chen et al. 2018), NC_036011.1 (Li et al. 2017), NC_037926.1 (Tan et al. 2018), NC_023083.1 (Wang et al. 2014).

Both maximum likelihood (ML) and Bayesian inference (BI) analyses recovered identical topological structures with strong support values at the majority of the major nodes (Figure 3). The monophyly of Reduviidae was strongly supported (BS = 100, PP = 1.00). The recovered subfamily relationships were as follows: Mirinae + (Notonectinae + (Phymatinae + (Peiratinae + (Harpactorinae + (Triatominae + Reduviinae))))).

Within Reduviidae, Reduviinae was strongly supported as a monophyletic group (BS = 100, PP = 1.00). *Acanthaspis geniculata* (PX310134.1) was stably placed within the Reduviinae clade. Within the Reduviinae, the phylogenetic analysis revealed the following topological relationships: (*A. geniculata* + (*A. ruficeps* [NC_037369.1]+ *A. pedestris* [NC_069624.1])) + (*A. cincticrus* [NC_037735.1]+ (*Inara alboguttata* [NC_037736.1]+ (*Reduvius gregoryi* [NC_037746.1]+ *Platymeris biguttatus* [PP198281.1]))).

## 5. Discussion and conclusions

In this study, we report the first complete mitochondrial genome of *Acanthaspis geniculata*, providing valuable mitogenomic resources for the Reduviinae subfamily. The mitogenome exhibits typical characteristics shared with other sequenced Reduviidae species, including a circular molecule of 16,226 bp in length, a pronounced AT bias (70%), which is primarily driven by mutation pressure and natural selection, and consequently favors synonymous codons ending with A or T (Li et al. 2017; Zhao et al. 2022). It comprises 37 mitochondrial genes (13 PCGs, 2 rRNAs, 22 tRNAs), and a control region. The D-loop (control region), spanning 1,395 bp and located between rrnS and tRNA-Ile, contains essential elements for the initiation of mitochondrial replication and transcription. Notably, as in most Reduviidae mitogenomes, an incomplete stop codon (a single ‘T’) was identified in the COX1 gene (Li et al. 2017; Zhao et al. 2019). Additionally, the mitogenome features highly conserved gene arrangements and specific gene overlaps (Gong et al. 2018; Zhao et al. 2022), such as the 7 bp overlap observed between ATP8/ATP6 and ND4/ND4L, aligning with the ancestral insect mitochondrial gene order. These overlaps are typical of insect mitochondrial genomes and are often considered to relate to genome size economy and post-transcriptional regulation. Unlike the complex tRNA rearrangements or duplications reported in other Reduviidae species (Jiang et al. 2016), *A. geniculata* shows no apparent tRNA shuffling, suggesting that the mitochondrial gene order in this species represents a highly conserved ancestral state within the Reduviinae subfamily.

At the phylogenomic level, both ML and BI analyses yielded identical and robust topologies, confirming that the classical morphology-based classification of Reduviidae is largely consistent with molecular evidence. The phylogenetic reconstruction clearly divided the ingroup into six well-supported subfamily clades, with the monophyly of Reduviidae (BS = 100, PP = 1.00) strongly corroborated.

Previous phylogenetic studies based on limited molecular markers (e.g. partial COI or nuclear ribosomal genes) often resulted in a poorly resolved or even non-monophyletic Reduviinae, mainly due to insufficient taxon sampling or insufficient phylogenetic signals (Hwang and Weirauch 2012; Bhagyasree 2019). In contrast, our analysis—which utilizes complete mitogenomes with optimized partitioning models and both ML and BI approaches—strongly supports Reduviinae as a monophyletic group (BS = 100, PP = 1.00). Within Reduviinae, *Acanthaspis geniculata* was recovered as a sister taxon to the clade comprising *A. ruficeps* and *A. pedestris*, while forming a larger clade with *A. cincticrus*, *Inara alboguttata*, *Reduvius gregoryi*, and *Platymeris biguttatus*.

Regarding the higher-level relationships within Reduviidae, our tree topologically inferred a sister-group relationship between Reduviinae and Triatominae with high support (UFBoot = 94.6, PP = 1.00), which is consistent with the previous findings (Weirauch and Munro 2009). However, it is important to note that the node grouping Harpactorinae with the (Reduviinae + Triatominae) clade received unexpectedly low support (UFBoot = 40.1, PP = 0.65). This weak signal suggests that while our mitogenome data robustly resolve the monophyly of Reduviinae and its close relationship with Triatominae, the exact placement of Harpactorinae remains poorly resolved. This uncertainty may be due to rapid evolutionary radiation or short internal branch lengths during the early divergence of these major subfamilies. Therefore, future research should focus on expanding genus-level sampling and incorporating nuclear genomic data to further clarify the basal relationships among the Reduviidae subfamilies.

## Supplementary Material

Supplemental Material

## Data Availability

The genome sequence data supporting this study’s findings are available in GenBank of NCBI at (https://www.ncbi.nlm.nih.gov) under assessment No. PX310134. The associated BioProject, SRA, and Bio-Sample numbers were PRJNA1321283, SRR35311901, and SAMN51202329, respectively.
